# Improvement of memory function via a combination of exercise and soy peptide supplementation in community-dwelling older adults: A randomized controlled trial

**DOI:** 10.1016/j.conctc.2022.100998

**Published:** 2022-09-09

**Authors:** Masakazu Imaoka, Hidetoshi Nakao, Misa Nakamura, Fumie Tazaki, Mitsumasa Hida, Ryota Imai, Motohiro Maebuchi, Masahisa Ibuki, Masatoshi Takeda

**Affiliations:** a Department of Rehabilitation, Osaka Kawasaki Rehabilitation University, 158 Mizuma, Kaizuka, Osaka, 597-0104, Japan; b Cognitive Reserve Research Center, 158 Mizuma, Kaizuka, Osaka, 597-0104, Japan; c Graduate School, Department of Comprehensive Rehabilitation, Osaka Prefecture University, 3-7-30 Habikino, Habikino, Osaka, 583-8555, Japan; d Department of Preventive Gerontology, National Center for Geriatrics and Gerontology, 7-430 Morioka, Obu, Aichi, 474-8511, Japan; e Strategy Planning Department, R&D Division, Fuji Oil Co., Ltd., 1-1 Sumiyoshi, Izumisano, Osaka, 598-8540, Japan; f Japan Nutrition Co.,Ltd. Shin-Aoyama Bldg. West Tower 22F, Tokyo, 107-0062, Japan

**Keywords:** Memory, Motor function, Dietary supplements, Elderly, MMSE, Mini-Mental State Examination, ACE-R, Addenbrooke's Cognitive Examination-Revised, Ex + Nt, exercise plus nutrition group, Ex, exercise-only group, GDS-15, Geriatric Depression Scale 15, SMI, skeletal muscle mass index, ANOVA, analysis of variance, SPI, soy protein isolate, BDNF, brain-derived neurotropic factor

## Abstract

**Background:**

Soy peptide, when consumed as a functional food, has been reported to improve cognitive function. This study aimed to verify the combined effect of soy peptide supplementation and exercise on cognitive function among community-dwelling older adults in Japan.

**Methods:**

In this population-based, non-blinded randomized controlled trial, 72 community-dwelling older adults who were independent in activities of daily living were randomly assigned to an “exercise plus nutrition” program (Ex + Nt group, n = 36) or an exercise program (Ex group, n = 36). For 3 months, both groups participated in an exercise and cognitive training regimen once per week, with the Ex + Nt group receiving soy supplementation once per week. Pre- and post-intervention measurements included grip strength, gait speed, skeletal muscle mass index, and scores on Addenbrooke's Cognitive Examination-Revised, trail-making test A, and the Geriatric Depression Scale. Participant enrollment for this study started in January 2019 and ended in April 2019.

**Results:**

Exercise training increased the skeletal muscle mass index by 2.0% and 3.0% in the Ex + Nt and Ex groups, respectively. The Ex + Nt group exhibited a significant 0.3-point increase in the memory score.

**Conclusion:**

A 3-month exercise program combined with soy peptide supplementation may be effective in improving both motor and memory function in community-dwelling older adults.

## Highlights

• A non-blinded randomized controlled trial in community-dwelling older adults.
• Weekly exercise program compared to soy supplementation plus exercise for 3 months.
• Skeletal muscle mass index increased in both groups.
• Soy peptide supplementation also increased memory scores.

## 1 Introduction

According to the Ministry of Internal Affairs and Communications, in 2020, Japan will have the highest aging rate worldwide, with the population of older adults aged ≥65 years reaching 36.17 million, accounting for 28.7% of the total population [1]. Furthermore, based on analyses of the Ministry of Health, Labour, and Welfare, the rate of aging is expected to increase until 2042, even if the population declines, with those aged ≥65 years reaching 38.4% of the total population by 2065 and resulting in a society with approximately 1 in 2.6 people being >65 years of age [1]. Accordingly, the prevalence of dementia and cognitive impairment is expected to gradually increase. The number of patients with dementia in 2012 was approximately 4.6 million, representing 15% of the older adult population, but it is estimated that by 2025, one in five older adults will have dementia. Alzheimer's dementia is the most common type of dementia. Diabetes, depression, hypertension, and genetics have been identified as risk factors for dementia [[2], [3], [4], [5]]. In addition, there are various social risk factors for dementia, such as inactivity and low income [6,7]. However, factors such as attaining higher education [8], habitual physical activity, engagement in intellectual activity, and intake of antioxidant-containing foods have been shown to protect against Alzheimer's disease [[8], [9], [10], [11]].

Exercise is a habit that can be started easily and is effective in maintaining and improving cognitive function [12]. Moreover, some exercises do not require any equipment or cost money. The effects of aerobic, strength, coordination, and mixed training have already been reported [13]. Among these, coordination training (e.g., dance) is said to be the most effective for improving cognitive function, and research has indicated that performing pre-memorized motions along with music may enhance attention and executive functions. The mechanism underlying the beneficial effect of exercise on brain function is believed to involve decreases in the risk of cerebrovascular disease, suppression of inflammation, strengthening of brain structure due to increases in brain growth factor levels, and reductions in amyloid accumulation [14]. At the biological level, improved insulin resistance leads to improved synaptic function and increased brain capacity, which is thought to contribute to improved cognitive function. Among the various types of exercise, aerobic training has been regarded as particularly effective, with reports recommending at least 45 min of aerobic exercise per session [15].

In recent decades, the link between nutritional habits and cognitive health has received growing attention. Undernutrition is associated with cognitive decline [16]. Moreover, some food components, such as docosahexaenoic acid, Ginkgo extract, polyphenols, and soy peptide, have been reported to improve cognitive function [[17], [18], [19], [20]]. Our research has focused on soy peptide, which has been shown to improve both brain and muscle function [21,22], and we have demonstrated that the combination of exercise and soy peptide intake can improve Mini-Mental State Examination (MMSE) scores in community-dwelling older adults [23]. The soybean peptide used at this time was a beverage called “peptide athlete,” which contained 4000 mg of soybean peptide in 250 g. However, this rating scale, which is mainly used in dementia screening tests, may be unsuitable for examining cognitive function in healthy older adults. Indeed, a previous study noted the ceiling effect of the MMSE for the evaluation of cognitive function in several groups of healthy people [24].

Therefore, in this study, we utilized Addenbrooke's Cognitive Examination-Revised (ACE-R) [[25], [26], [27]] test of general cognitive function to verify the effects of exercise combined with soy peptide supplementation on cognition in healthy older adults. In addition, we evaluated the effect of this combination on muscle mass when compared with exercise alone.

## 2 Material and methods

Participants included community-dwelling older adults aged ≥60 years from an area of Osaka, Japan, who were independent in their activities of daily living. Participant enrollment for this study started in January 2019 and ended in April 2019. Those with doctor's orders to stop exercise, those who were medically advised not to participate in exercise, those with dementia, and those with missing pre-intervention data were excluded. This non-blinded randomized controlled trial evaluated the effects of soy peptide supplementation in conjunction with a multicomponent exercise program (multiple forms of exercise that combined music and cognitive training) on cognitive function in older adults, which were compared with the effects of exercise alone [23]. The Osaka Kawasaki Rehabilitation University Ethics Committee approved the trial protocol (approval number OKRU29-A021). The trial was registered in the University Hospital Medical Information Network Clinical Trial Registry (registration number UMIN000034984). In accordance with the tenets of the Declaration of Helsinki, all participants provided written informed consent for study participation, and no stipend was provided. Randomization was performed by generating random numbers using a computer, to adjust for confounding factors. Based on rounding, participants with values ≥ 0.5 were assigned to the exercise plus nutrition group (Ex + Nt, n = 36), while those with values < 0.5 were assigned to the exercise-only group (Ex, n = 36).

A multicomponent exercise program was performed at the Kaizuka City Welfare Center. Participants engaged in 15 min of memory training and 45 min of aerobic exercise once per week for 3 months. As part of the memory training session, participants listened to a novel sentence, repeated it, and performed two tasks: stepping on a designated spot and clapping their hands in multiples of three and five times. The aerobic exercise session included 10 min of self-stretching followed by 15 min of aerobic exercise therapy. This was followed by conditioning for 10 min to normalize the heart rate. The aerobic exercise session included calisthenics performed to a song by the locally popular character “Tsugasan.” Gymnastic activities were performed on a yoga mat (60 cm × 173 cm). The regimen included lower limb steps, upper limb rhythmic movements, finger movements, and a 10-min mindfulness exercise in the recumbent position. All exercises were taught by physiotherapists and staff volunteers. In addition, all participants were provided an activity meter to self-monitor their daily number of steps. The activity meter was worn at all times for 3 months except when sleeping during the exercise class. The purpose of wearing the activity meter was to increase the habitual walking time by self-monitoring. Commercially available beverages (Peptide Athleeta 4000, Fuji Oil Co., Ltd., Osaka, Japan) were used as nutrition support to provide soy peptide supplementation in the Ex + Nt group. Table 1 shows the nutrition facts label for Peptide Athleeta 4000. The net weight of Peptide Athleeta 4000 containing 4 g of soy peptide was 190 g. One bottle was consumed every day during the exercise class, and the timing of drinking was not specified.

**Table 1:** Total calories and chemical composition of soybean.

Total calories (kcal)	48
Protein (g)	4.4
Fat (g)	0
Carbohydrate (g)	8.9
Sodium (mg)	112
Valine (mg)	184
Leucine (mg)	300
Isoleucine (mg)	177
Soybean peptide (mg)	4000

The primary outcome was cognitive status as measured using the Japanese version of the ACE-R [[25], [26], [27]]. The trail-making test, a well-known psychomotor test [28], was used to evaluate attentiveness. In addition, gait speed [29], grip strength [30], skeletal muscle mass index (SMI) [31] and the Geriatric Depression Scale 15 (GDS-15) [32] were also measured.

The ACE-R stands for Addenbrooke's Cognitive Examination Revised and is a simple cognitive function test that evaluates five cognitive areas: attention and orientation, memory, verbal fluency, language, and visuospatial cognition. It can be evaluated in 15–20 min, and the score is 100 points including MMSE in the test. In the Japanese version, 89 points or more are normal, 83–88 points are mild cognitive impairment, and 82 points or less are dementia.

For gait speed participants were instructed to walk 6.4 m (divided into two 2-m zones at each end and a 2.4-m zone in the middle) at a speed they found comfortable. The time needed (s) to pass the 2.4-m middle zone was measured to calculate the gait speed (m/s). We used the average of five gait trials. Grip strength was measured by handgrip strength, which has been reported to be significantly associated with whole-body muscle strength. The maximum voluntary isometric strength of the handgrip was measured using a grip-D hand dynamometer (Takei; Niigata, Japan) for the dominant hand while in a standing position. Physiological parameters measured using bioelectrical impedance analysis (InBody270; InBody, Tokyo, Japan) were obtained from the participants’ electronic medical records. The limb SMI was calculated as the limb skeletal muscle mass (kg) divided by the height squared (m^2^). GDS-15 is the mostly used screening test for depression worldwide. It consists of 15 short questions, and the subject answers “yes” or “no”, so it is easy to answer, easy to score, and the enforcement time was 5–7 min. A score of 5 or higher is rated as depressive trend, and a score of 10 or higher is rated as depressed.

Detectability calculations were performed to determine the sample size required to detect significant interaction effects between intervention and time (before and after), using the following parameters: medium effect size, 0.25; α error, 0.05; and β error, 0.20. The number of participants required was 33 in each group. The dropout rate was estimated to be 10% or higher; thus, the target number of participants was 36 per group.

All statistical analyses were performed using IBM SPSS Statistics for Windows version 25.0 (IBM Corp., Armonk, NY). A p-value <0.05 was considered statistically significant. Analysis of variance (ANOVA) was used for intra- and inter-group comparisons of continuous variables, and the chi-squared test was used for inter-group comparisons of categorical variables at baseline. In addition, the effect of each intervention on the outcome measures was analyzed using a mixed 2 × 2 group (Ex, Ex + Nt group) × time (pre, post) two-way ANOVA. In the sub-analysis, the ACE-R orientation time, orientation location, registration, attention and calculation, recall and language, and practice sub-items were analyzed separately for the Ex and Ex + Nt groups and compared using two-way ANOVA.

## 3 Results

**Fig. 1:**
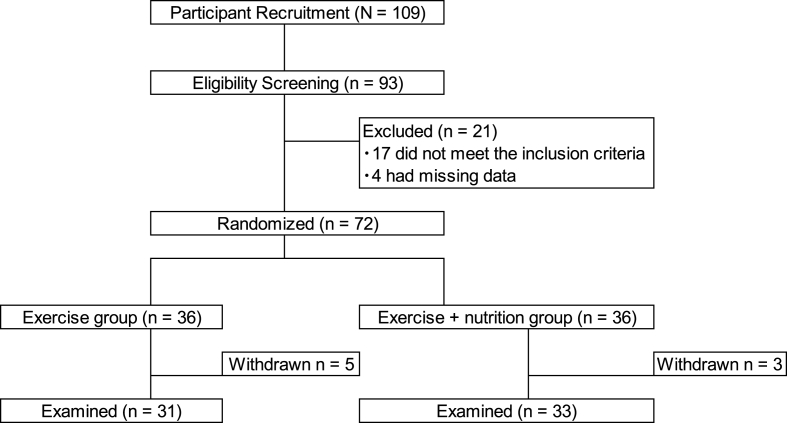
Flowchart of trial participation.

Of the 72 participants included in the study, 64 (88.8%) completed the 3-month follow-up: 31 (86.1%) in the Ex group and 33 (91.7%) in the Ex + Nt group (Fig. 1). There was no significant difference in the baseline characteristics between the two groups (Table 2). The median dietary supplement adherence rate was 90%, and the participation rate in the exercise session was ≥90%. No adverse events (health problems, musculoskeletal complications, muscle aches, or falls) were identified during the intervention period.

**Table 2:** Baseline characteristics of the study participants.

Characteristics	Overall (n = 72)	Ex + Nt group (n = 36)	Ex group (n = 36)	F-value	p-value
Age	74.7 ± 5.1	74.7 ± 4.8	74.7 ± 5.4	1.233	1.000
Height, cm	154.6 ± 7.3	153.8 ± 7.8	155.3 ± 6.7	0.606	0.405
Weight, kg	53.5 ± 8.9	52.6 ± 8.9	54.5 ± 9.0	0.002	0.308
Females, n %	59 (81.9%)	28 (77.8%)	31 (86.1%)		0.271
ACE-R, score	91.5 ± 6.7	90.5 ± 7.4	92.6 ± 4.8	1.416	0.203
TMT-A, sec	102.1 ± 37.1	105.3 ± 38.2	96.8 ± 33.3	0.026	0.217
Gait speed, m/s	1.40 ± 0.20	1.37 ± 0.17	1.43 ± 0.23	2.260	0.271
Grip strength, kg	23.3 ± 6.0	23.3 ± 5.5	23.3 ± 6.7	0.936	0.947
SMI, kg/m^2^	5.94 ± 0.90	5.93 ± 0.87	5.95 ± 0.93	0.893	0.805
GDS, score	3.1 ± 2.6	2.8 ± 2.4	3.3 ± 2.7	0.171	0.667

Notes**:** Data are presented as the mean ± standard deviation or numbers (percentages). **Abbreviation**s**:** Ex group, participants allocated to an exercise program; Ex + Nt group, participants allocated to a combined exercise and soy peptide intake program.

The pre- and post-intervention group statistics and group-time interactions are shown in Table 3. Significant time-specific effects were observed for the SMI. Exercise training resulted in an increase in the SMI in both groups, by 2.0% in the Ex + Nt group and by 3.0% in the Ex group. Table 4 presents the changes in the cognitive domain, task, and subtotal scores of the ACE-R in the Ex and Ex + Nt groups. The Ex + Nt group exhibited an improvement of 0.3 points in the memory domain score, indicating a statistically significant interaction between time and group (p < 0.05).

**Table 3:** Pre- and post-intervention outcome measurements in the two groups.

	Pre-intervention		Post-intervention	Two-way ANOVA			
				Time Effect		Time × Group Interaction	
				F-value	p-value	F-value	p-value
ACE-R (score)							
	Ex + Nt	90.5 ± 7.4	90.2 ± 8.9	1.78	0.19	0.30	0.59
	Ex	92.6 ± 4.8	92.0 ± 6.4				
TMT-A (s)							
	Ex + Nt	105.3 ± 38.2	104.0 ± 39.7	0.30	0.59	0.81	0.37
	Ex	96.8 ± 33.3	88.7 ± 24.7				
Gait speed (m/s)							
	Ex + Nt	1.37 ± 0.17	1.34 ± 0.17	2.25	0.14	0.00	0.97
	Ex	1.43 ± 0.23	1.42 ± 0.17				
Grip strength (kg)							
	Ex + Nt	23.3 ± 5.5	23.3 ± 6.1	1.25	0.27	2.51	0.12
	Ex	23.3 ± 6.7	24.7 ± 6.7				
SMI (kg/m^2^)							
	Ex + Nt	5.93 ± 0.87	6.04 ± 0.93	21.20	<0.05*	0.36	0.55
	Ex	5.95 ± 0.93	6.13 ± 0.90				
GDS (score)							
	Ex + Nt	2.8 ± 2.4	2.40 ± 2.12	3.27	0.08	0.77	0.40
	Ex	3.3 ± 2.7	3.00 ± 2.90				

Notes**:** Data are presented as the mean ± standard deviation.

**Abbreviation**s**:** Ex group, participants allocated to an exercise program; Ex + Nt group, participants allocated to a combined exercise and soy peptide intake program; ACE-R, Addenbrooke's Cognitive Examination-Revised; TMT, Trail-making test; IGF-1, insulin-like growth factor 1; SMI, skeletal muscle mass index; GDS, Geriatric Depression Scale.

*p < 0.05.

**Table 4:** Pre- and post-intervention ACE-R cognitive domain, task, and subtotal scores.

		Pre-intervention	Post-intervention	Two-way ANOVA			
				Time Effect		Time × Group Interaction	
				F-value	p-value	F-value	p-value
Attention							
	Ex + Nt	17.7 ± 0.6	17.5 ± 1.2	0.66	0.42	1.11	0.30
	Ex	17.8 ± 0.4	17.8 ± 0.4				
Memory							
	Ex + Nt	21.2 ± 4.3	21.5 ± 4.5	1.89	0.18	4.53	<0.05*
	Ex	22.7 ± 3.1	21.3 ± 3.8				
Fluency							
	Ex + Nt	12.3 ± 2.2	11.6 ± 2.8	2.28	0.14	1.14	0.29
	Ex	12.9 ± 1.5	12.8 ± 2.1				
Language							
	Ex + Nt	24.3 ± 1.9	24.5 ± 1.7	2.51	0.12	0.12	0.74
	Ex	24.3 ± 1.4	24.6 ± 1.7				
Visuospatial							
	Ex + Nt	15.2 ± 1.1	15.1 ± 1.1	1.64	0.21	2.41	0.13
	Ex	15.4 ± 0.7	15.8 ± 0.6				

Notes**:** Data are presented as the mean ± standard deviation.

**Abbreviations:** Ex group, participants allocated to an exercise program; Ex + Nt group, participants allocated to a combined exercise and soy peptide intake program; ACE-R, Addenbrooke's Cognitive Examination-Revised. *p < 0.05.

## 4 Discussion

In this study, we examined the effect of combined exercise intervention and soy peptide supplementation in community-dwelling older adults. The improvement effect of SMI was confirmed in both Ex and Ex + Nt groups. On the other hand, only the Ex + Nt group that ingested soy peptide showed significant improvement in the items related to memory of cognitive function.

Memory function and muscle mass decrease with increasing age [33,34]. Studies have revealed that cognitive function begins to decline around the age of 60 years, with calculation and memory being especially affected by aging. Therefore, intervention programs that can effectively improve cognitive function, muscle mass, and muscle strength in community-dwelling older adults within a short period of time are required. Our study, which was based on assessments of cognitive function using the ACE-R, indicates that combined exercise and nutritional supplementation can improve memory function in older adults.

The soy peptide supplement used for nutrition intervention was made from enzyme digests of soy protein isolate (SPI). The protein digestibility-corrected amino acid score of the SPI is 1.0, the highest possible score [35]. This indicates that the soy peptide produced from SPI has an ideal amino acid balance. Soy peptide has been reported to exert beneficial effects on muscle and brain functions [21,22], with the effect on muscles being attributed to rapid absorption in the small intestine [36]. Previous studies have also reported that soy peptide intake can improve cognitive abilities such as sustained attention and short- and long-term memory in healthy adults [17,21]. These effects may be related to the actions of physiological functional peptides, such as Ser-Tyr and Gly-Arg, which upregulate norepinephrine and brain-derived neurotropic factor (BDNF) in the brain [[36], [37], [38]]. The possibility of naturally occurring peptides passing through the blood-brain barrier has been examined in recent rodent studies, which have demonstrated that dipeptide-containing soy peptide crosses the blood-brain barrier and exerts an ameliorative effect on cognitive dysfunction [39,40].

In our study, soy peptide intake in combination with the multicomponent exercise intervention was found to improve composite cognitive function, although exercise alone contributed to selective improvement in general cognitive function. Previous studies have demonstrated that physical exercise increases BDNF levels [41]. Thus, differences in the effects of each program on cognitive function may have occurred because increases in brain growth factor levels (BDNF, insulin-like growth factor 1, and vascular endothelial growth factor) were more likely to occur in the Ex + Nt group than in the Ex group [14]. We speculated that soy peptide may have been positively involved in the underlying mechanism.

We also observed improvements in muscle mass in the present study, with a 3.0% increase in the Ex group and a 2.0% increase in the Ex + Nt group. Previous studies involving older adults engaged in 3 months of exercise intervention have reported conflicting results regarding improvements in muscle mass [[42], [43], [44]]. This may be largely due to differences in the programs implemented and the frequency of exercise. Although the frequency of exercise was low in our study (once per week), improvements in muscle mass may have occurred given that the program consisted of simple exercises that could be performed voluntarily at home. In addition, previous studies have revealed that the amount of activity increases more than usual when an activity meter is worn, and it is possible that the increase in the number of daily steps contributed to the increase in muscle mass [45]. The improvement in muscle mass in both groups proved to be significant even with a single weekly 60-min exercise class, which is consistent with the results of our preliminary study [23]. Since we also encouraged voluntary exercise on days when exercise classes were not held, we need to further verify how efforts other than the exercise classes affected body composition.

Our study had some limitations. First, dietary and fluid intake were not recorded. Therefore, we could not eliminate the effects of variations in nutritional intake and uncontrolled calorie intake. Second, the exercise classes were held only once a week, and the intensity and frequency of the exercises performed on other days were unclear. Third, since all participants were community-dwelling older adults in Japan, care must be taken when generalizing the survey results to other ethnic groups. Finally, it remains unclear to what extent the effects of the combined intervention can be sustained. In order to better explain the physiological results, future studies should include various populations, blood testing, and evaluation of the long-term effects of combined exercise and nutritional supplementation.

## 5 Conclusion

Three months of weekly multicomponent exercise alone or in combination with soy peptide supplementation can improve memory and physical functions in community-dwelling older adults. In addition to improving motor function, the combination of exercise and nutritional supplementation can improve memory function in this population.

## Funding

This work was supported by Fuji Oil Co., Ltd. and Osaka Group Welfare Foundation. The sponsors had no role in the design and conduct of the study; in the collection, analysis, and interpretation of data; in the preparation of the manuscript; or in the review or approval of the manuscript.

## Author information

Masakazu Imaoka is an Assistant Professor at Osaka Kawasaki Rehabilitation University, Japan. His work focuses on frailty, sarcopenia, and fall prevention in older adults. He has a master's degree and PhD in Health Sciences from Osaka Prefecture University.

## Authors’ contributions

MI, HN, FT, MH and RI performed data measurements, and MI, MN and MT performed data analysis. MI and MM provided grant support. MI was a major contributor to the manuscript drafting. All authors read and approved the final manuscript.

## Declaration of competing interest

The authors declare that they have no known competing financial interests or personal relationships that could have appeared to influence the work reported in this paper.

## Data Availability

Data will be made available on request.
